# Relationship between the Number of Repeats in the Neck Regions of L-SIGN and Augmented Virus Replication and Immune Responses in Dengue Hemorrhagic Fever

**DOI:** 10.3390/ijms25105497

**Published:** 2024-05-17

**Authors:** Keh-Sen Liu, Po-Ming Chen, Lin Wang, Ing-Kit Lee, Kuender D. Yang, Rong-Fu Chen

**Affiliations:** 1 Division of Infectious Diseases, Department of Internal Medicine, Show Chwan Memorial Hospital, Changhua 500, Taiwan; milka2@msn.com; 2 Research Assistant Center, Show Chwan Memorial Hospital, Changhua 500, Taiwan; yaoming9@yahoo.com.tw; 3 Department of Nursing, College of Health Sciences, Central Taiwan University of Science and Technology, Taichung 406, Taiwan; 4 Department of Pediatrics, Pojen Hospital, Kaohsiung 813, Taiwan; 5 Division of Infectious Diseases, Department of Internal Medicine, Chang Gung Memorial Hospital-Kaohsiung Medical Center, Chang Gung University College of Medicine, Kaohsiung 833, Taiwan; 6 Departments of Medical Research, MacKay Memorial Hospital, Taipei 104, Taiwan; 7 Departments of Pediatrics, MacKay Memorial Hospital, Taipei 104, Taiwan; 8 Department of Medicine, MacKay Medical College, New Taipei 252, Taiwan; 9 Division of Plastic Surgery, Department of Surgery, Kaohsiung Medical University Hospital, Kaohsiung 807, Taiwan; 10 Regenerative Medicine and Cell Therapy Research Center, Kaohsiung Medical University, Kaohsiung 807, Taiwan

**Keywords:** L-SIGN, tandem repeats, dengue hemorrhagic fever, T-helper 2 cells

## Abstract

C-type lectins play a crucial role as pathogen-recognition receptors for the dengue virus, which is responsible for causing both dengue fever (DF) and dengue hemorrhagic fever (DHF). DHF is a serious illness caused by the dengue virus, which exists in four different serotypes: DEN-1, DEN-2, DEN-3, and DEN-4. We conducted a genetic association study, during a significant DEN-2 outbreak in southern Taiwan, to explore how variations in the neck-region length of L-SIGN (also known as CD209L, CD299, or CLEC4M) impact the severity of dengue infection. PCR genotyping was utilized to identify polymorphisms in variable-number tandem repeats. We constructed L-SIGN variants containing either 7- or 9-tandem repeats and transfected these constructs into K562 and U937 cells, and cytokine and chemokine levels were evaluated using enzyme-linked immunosorbent assays (ELISAs) following DEN-2 virus infection. The L-SIGN allele 9 was observed to correlate with a heightened risk of developing DHF. Subsequent results revealed that the 9-tandem repeat was linked to elevated viral load alongside predominant T-helper 2 (Th2) cell responses (IL-4 and IL-10) in K562 and U937 cells. Transfecting K562 cells in vitro with L-SIGN variants containing 7- and 9-tandem repeats confirmed that the 9-tandem repeat transfectants facilitated a higher dengue viral load accompanied by increased cytokine production (MCP-1, IL-6, and IL-8). Considering the higher prevalence of DHF and an increased frequency of the L-SIGN neck’s 9-tandem repeat in the Taiwanese population, individuals with the 9-tandem repeat may necessitate more stringent protection against mosquito bites during dengue outbreaks in Taiwan.

## 1. Introduction

Dengue fever (DF), resulting from the dengue virus (DEN), is widespread in tropical and subtropical regions, with an estimated minimum of 50 million new cases annually. The severe manifestations, dengue hemorrhagic fever (DHF) and dengue shock syndrome (DSS), pose significant threats to populations across nearly two-thirds of the world’s countries, particularly in Southeast Asia [1]. Dengue has the ability to bind to lectin-like receptors, leading to the release of cytokines and other immune response mediators [2,3]. DC-SIGN (dendritic-cell-specific ICAM-3 grabbing nonintegrin, encoded by CD209) has been demonstrated to serve as a crucial dengue receptor located on the surface of dendritic cells derived from human monocytes [4]. More recently, it has been discovered that L-SIGN (liver/lymph node-specific ICAM-3 grabbing nonintegrin, encoded by CLEC4M, also known as CD299) can also engage with the dengue virus. [5,6,7]. DC-SIGN has been demonstrated to facilitate intracellular signaling pathways, resulting in the secretion of cytokines, and to exploit this mechanism as a component of dengue virus’s immune evasion tactics [8]. Increasing evidence suggests that the host’s genetic factors play a significant role in determining variations in susceptibility to dengue infection and may influence the clinical manifestation of the disease [9]. 

L-SIGN, a homolog of DC-SIGN, shares 77% amino acid identity. Unlike DC-SIGN, primarily found on dendritic cells, L-SIGN is expressed in the liver, lymph nodes, and placenta [10,11,12]. Both DC-SIGN and L-SIGN share the ability to bind high-mannose oligosaccharides through their carbohydrate recognition domain and have been shown to recognize a vast range of microbes, such as HIV-1, Ebola, HCV, severe acute respiratory syndrome-associated coronavirus (SARS)-CoV, and Mycobacterium tuberculosis [11,13,14]. DEN-2 generated in primary dendritic cells (DCs) lacks the ability to engage with DC-SIGN but retains infectivity for cells expressing L-SIGN [15]. The neck region participates in assembling both lectins into a tetrameric protein conformation on the cell surface. The length of this neck region may potentially influence the pathogen-binding properties of these lectin receptors [6]. Studies have investigated the associations between the highly polymorphic variable-number tandem repeats (VNTRs) of the neck region in L-SIGN and host’s susceptibility to various infectious diseases, including HIV-1, SARS, and HCV [14,16,17]. 

Therefore, we hypothesized that length variations in the DC-SIGN and L-SIGN neck regions might affect individual susceptibility to DF and/or DHF. To test this hypothesis, we investigated the relationship between the DC-SIGN and L-SIGN tandem repeat variations in the neck region and the susceptibility to DF and DHF in a large cohort of southern Taiwan origin. Employing a hospital-based DHF vs. DF case-control study, we studied the relationship of the severity of dengue infections and/or the DEN-2 virus load to the host’s genetic DC-SIGN and L-SIGN neck repeat polymorphisms. Clarification of the relationships between the host’s genetic background and the severity of dengue infection with DHF and/or the virus load and cytokines in the blood may provide better insight for the prevention and treatment of DHF. 

We proposed that variations in the length of the neck regions of DC-SIGN and L-SIGN may influence individual susceptibility to DF and/or DHF. To investigate this hypothesis, we examined the association between variations in the tandem repeats of the neck regions of DC-SIGN and L-SIGN and susceptibility to DF and DHF in a large cohort from southern Taiwan. Through a hospital-based case-control study focusing on DHF versus DF, we explored the correlation between the severity of dengue infections and/or the DEN-2 virus load and genetic polymorphisms in the neck regions of DC-SIGN and L-SIGN. Elucidating the interplay between the host’s genetic factors, disease severity, virus load, and cytokine levels in the blood may offer valuable insights for the prevention and treatment of DHF.

## 2. Results

### 2.1. Clinical Symptoms and Warning Signs in DHF and DF Groups

This study used a case-control design to compare two groups of patients with DHF or DF in a DEN-2 outbreak between 2002 and 2003 in Taiwan. DEN-2 infection was defined by the presence of compatible symptoms and signs in patients with detectable DEN-2 RNA by quantitative RT-PCR or the detection of IgM specific for DEN in their blood samples [18,19]. The diagnosis of DHF was made according to the criteria of the World Health Organization for DHF [20,21]. In a case-control study, we matched 104 DF and 109 DHF patients to 104 patients with OFI as a control group for studying the association of neck length variations in DC-SIGN and/or L-SIGN with DF and DHF. Analysis of demographic data found no significant difference between patients with DHF and DF with respect to gender and total leukocyte counts. In contrast, platelet counts; serum albumin, aspartate transaminase (AST), and alanine transaminase (ALT) levels; plasma leakage, including pleural effusion, ascites, or hemoconcentration; and secondary infection rates differed significantly between patients with DHF and DF (Table 1). With regard to L-SIGN, which is known to express on liver sinusoidal endothelial cells and in lymph nodes [10,22,23], we found that DHF patients had significantly higher AST and ALT levels, suggesting liver involvement of DEN infection in DHF [24]. Genetic variations in L-SIGN may affect DEN replication and immune responses, resulting in the coagulopathy and vascular leakage that are characteristics of DHF. 

### 2.2. Frequency of L-SIGN Neck-Region Nine-Tandem Repeats Was Higher in Asians than in Africans and Caucasians

The DC-SIGN- and L-SIGN 69-nucleotide tandem repeats in exon 4 were genotyped by PCR followed by gel electrophoresis, and the results were further verified by DNA sequence analysis in selective cases of representative genotypes. In our study population, no genetic polymorphism was detected in the DC-SIGN neck region in a pilot study, with only the homozygous 7/7 genotype being observed. Four kinds of neck polymorphisms were documented within exon 4 of the L-SIGN neck region, showing from 5-, 6-, 7-, and 9-tandem repeats in length in the studied population. The frequencies of the tandem repeats of a total of 317 study participants were 12.9% for the 9-repeat allele, 68.5% for the 7-repeat allele, 3.8% for the 6-repeat allele, and 14.8% for the 5-repeat allele (Table 2). The L-SIGN allele and genotype distributions in Chinese [11] were similar to those in East Asians (predominantly Chinese) [25]. In contrast, the L-SIGN neck-region tandem-repeat allele and genotype distributions in Caucasian and African populations [13,26] were distinctly different from those in the Chinese and Taiwanese [11] (Table 2).

### 2.3. Frequency of L-SIGN Neck-Region Nine-Tandem Repeats Was Higher in DHF than in DF Patients

The 9-repeat allele was present in 20% of the 104 patients with DHF, as compared with 10% of the 109 patients with DF (*p* = 0.004, OR = 2.30, 95% CI = 1.31–4.05) and 10% of the 104 OFI controls (*p* = 0.004, OR = 2.31, 95% CI = 1.30–4.10; Table 3). Although the 7-repeat allele was found to be the lowest in frequency in DHF patients (64%) when compared with DF patients (71%, *p* = 0.093) and OFI controls (71%, *p* = 0.118), no significant differences were found. Nine genotypes were found in the L-SIGN repeat region in this study, as shown in Table 4. The frequencies of the 9/9, 9/7, 9/6, and 9/5 genotypes were higher in DHF (5%, 21%, 2%, and 7%) than in DF patients (3%, 12%, 0%, and 2%) but did not reach statistical significance. When the heterozygous 7/6 genotype was compared between DHF patients (1%) and OFI controls (9%), a significant difference was found (*p* = 0.032, OR = 0.10, 95% CI = 0.01–0.82). Overall, these results suggested that the 9-tandem repeat allele carries a higher risk for DHF, and the heterozygous 7/6 genotype tends to be protective from DHF.

### 2.4. Increased DEN-2 Replication and Immune Inhibitions in DHF Patients with L-SIGN Neck-Region Nine-Tandem Repeats

**Figure 1:**
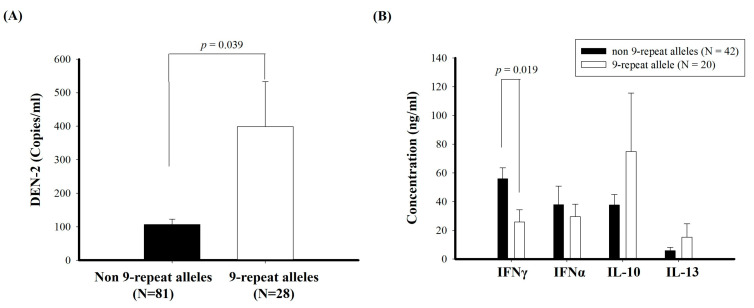
Dengue virus-2 (DEN-2) RNA copies (**A**) and Th1/Th2 cytokines (**B**) in blood from dengue patients with 9-repeat alleles and with non-9-repeat alleles. Data represent the means. Bars represent SEs.

Because the neck-region 9-tandem repeat allele was associated with DHF susceptibility, we analyzed whether the 9-repeat allele vs. the non-9-repeat alleles might affect the viral load and/or immune responses. We examined the viral load by RT-PCR, and measured Th1/Th2 cytokine levels (IFN-α, IFN-γ, IL-10, and IL-13) in the blood. Patients with the neck-region 9-repeat allele had a significantly higher DEN-2 viral load in the blood compared with those with the non-9-repeat alleles (398.9 ± 133.8 copies/μg RNA vs. 106.6 ± 16.5 copies/μg RNA; *p* = 0.039, Figure 1A). In contrast, patients with the neck-region 9-repeat allele had significantly lower IFN-γ levels (25.93 ± 8.43 pg/mL) than those with the non-9-repeat alleles (53.90 ± 7.57 pg/mL, *p* = 0.019, Figure 1B). There was no significant difference in the IFN-α, IL-10, or IL-13 levels between patients with the neck-region 9-repeat allele vs. the non-9-repeat alleles. Our report demonstrated that humans who bear the L-SIGN 9-tandem repeat allele are at higher risk of the development of DHF and tend to have depressed IFN-γ levels with higher viral loads once they are infected with DEN.

### 2.5. Increased DEN-2 Replication and Immune Inhibitions in K562 Cells with L-SIGN Neck-Region Nine-Tandem Repeats

**Figure 2:**
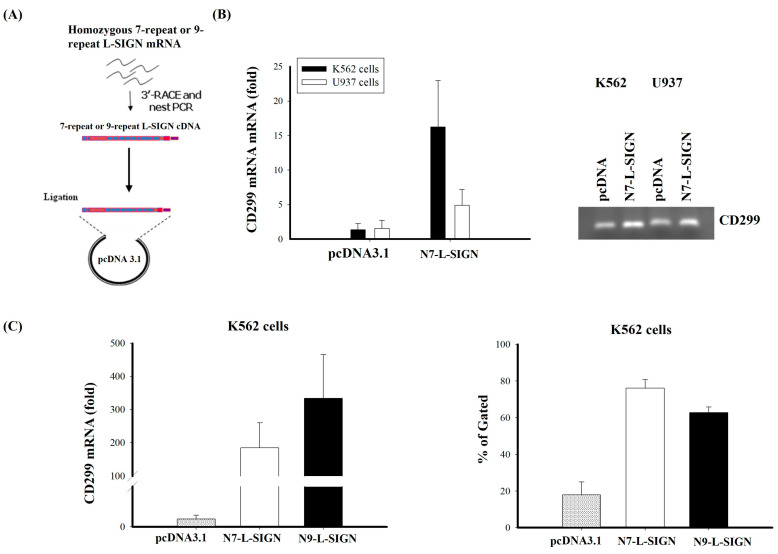
K562 and U937 cells were transfected with pcDNA3.1-N7-L-SIGN (7-repeat) and pcDNA3.1-N9-L-SIGN (9-repeat) plasmids for 48 h and assessed by q-RT-PCR analyses. (**A**) Strategy of molecular cloning in the construction of pcDNA3.1-N7-L-SIGN and pcDNA3.1-N9-L-SIGN plasmids. (**B**) Expression of N7-L-SIGN mRNA in parental (PR) and transfectants (TFs) of K562 and U937 cells by q-RT-PCR analyses and agarose gel electrophoresis. (**C**) Expressions of N7-L-SIGN and N9-L-SIGN mRNA assessed by q-RT-PCR analyses (n = 6), and percentage of gated cells analyzed by flow cytometry (n = 5) in parental and transfectants (N7 or N9) of K562.

**Figure 3:**
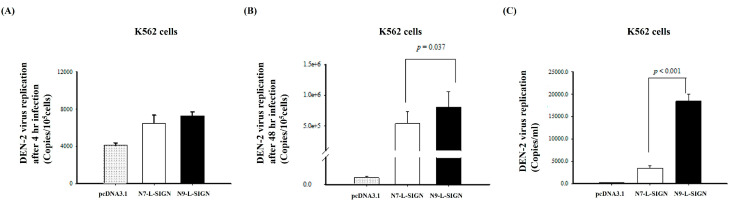
(**A**) Virus inputs and (**B**) intracellular and (**C**) extracellular viral loads of DEN-2 in L-SIGN transfected K562 cells. The virus titers in intracellular parts and supernatants were determined by TaqMan fluorogenic RT-PCR. (**A**) The virus titers in intracellular parts collected at 4 h post infection, representing virus entry, are shown as follows: parental K562: 1335.2510 ± 280.8680 copies/10^5^ cells; N7: 2498.6693 ± 290.2611 copies/10^5^ cells; N9: 2614.4835 ± 291.6596 copies/10^5^ cells. (**B**) The virus titers in intracellular parts collected at 48 h post infection, representing virus replication, are shown as follows: parental K562: 12,397.6 ± 2414.6 copies/10^5^ cells; N7: 540,923.7 ± 195,331.7 copies/10^5^ cells; N9: 809,386.2 ± 247,964.9 copies/10^5^ cells, *p* = 0.037. (**C**) The virus titers in supernatants collected at 48 h post infection, representing virus release, are shown as follows: parental K562: 254.9 ± 55.5 copies/mL; N7: 3508.5 ± 457.3 copies/mL; N9: 18,451.6 ± 1632.7 copies/mL, *p* < 0.001. Data are presented as mean ± SE calculated from five pairs of samples collected at 4 h or 48 h.

L-SIGN is mainly expressed on liver sinusoidal endothelial cells; it was hard to isolate liver sinusoidal endothelial cells from patients with the 9- or non-9-repeat alleles. DC-SIGN is highly expressed on immature monocyte-derived dendritic cells Human whole blood is also widely used for the investigation of immune responses and cell effects in vitro. To investigate the roles of different L-SIGN tandem repeat alleles in DEN virus entry, replication, and immune responses, we transfected the 9-repeat allele (N9, where N denotes ‘neck’) and 7-repeat allele (N7) in monocytic cell lines using molecular cloning (Figure 2A). Stable clones of U937 and K562 cells revealing N7 L-SIGN expression were selected with 400 μg/mL of G418 for 2 to 3 weeks. As shown in Figure 2B, we found that K562 cells had more L-SIGN mRNA expression than U937 cells, so K562 cells were selected as a stable transfectant of L-SIGN for further study. Stable clones of K562 cells revealed N9 L-SIGN mRNA expression (Figure 2C, left panel) but had less L-SIGN^+^ gated cells than stable N7 K562 transfectants (Figure 2C, right panel). K562 cells were incubated for one hour in 24-well plates with supernatants containing DEN virus at a multiplicity of infection (MOI) of 5. The infectivity of the parental K562 cells with DEN virus was relatively lower than transfectants with the L-SIGN 7-tandem repeat (N7) and L-SIGN 9-tandem repeat (N9). To reflect the replication of DEN-2, we measured the virus load in K562 cells with or without the L-SIGN 7-tandem repeat or 9-tandem repeat at 48 hours post infection of DEN-2. The results showed that there was no difference in the virus input among the parental and L-SIGN 7-tandem repeat or 9-tandem repeat transfectants (Figure 3A). Transfectants with the L-SIGN 9-tandem repeat had a significantly higher intracellular virus load than those with the L-SIGN 7-tandem repeat and parental K562 cells (parental: 12,397.6 ± 2414.6 copies/10^5^ cells; N7 variant: 540,923.7 ± 195,331.7 copies/10^5^ cells; N9 variant: 809,386.2 ± 247,964.9 copies/10^5^ cells, *p* = 0.037; Figure 3B). Particularly, transfectants with the L-SIGN 9-tandem repeat had a significantly higher extracellular virus load than those with the L-SIGN 7-tandem repeat and parental K562 cells (parental: 254.9 ± 55.5 copies/mL; N7: 3508.5 ± 457.3 copies/mL; N9: 18,451.6 ± 1632.7 copies/mL, *p* < 0.001; Figure 3C). Our data indicate that the N9 variant of L-SIGN has enhanced DEN-2 replication compared to the N7 variant of L-SIGN and parental K562 cells.

**Figure 4:**
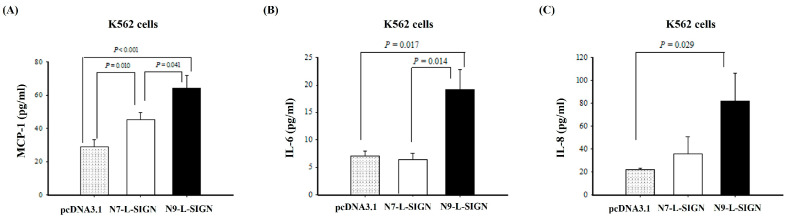
Production of (**A**) MCP-1, (**B**) IL-6, and (**C**) IL-8 in DEN-2-infected K562 cells. Data are presented as mean ± SE calculated from five pairs of samples collected in supernatants at 48 h post infection.

Higher plasma levels of IL-6, IL-8, MCP-1, IL-13, IL-18, TNF-α, IFN-α, and IFN-γ have been found in patients with severe DEN infections; we further compared the cytokine profiles among the parental and L-SIGN 7-tandem repeat or 9-tandem repeat transfectants. As shown in Figure 4A, it was found that K562 cells with 9-tandem repeat had higher MCP-1 levels than those with 7-tandem repeat transfectants or parental K562 cells (parental: 29.1 ± 4.1 pg/mL; N7: 45.4 ± 3.9 pg/mL; N9: 64.2 ± 7.6 pg/mL, respectively; *p* = 0.010, 0.041, <0.001). A comparison among parental K562 and L-SIGN 7-tandem repeat or 9-tandem repeat transfectants showed that K562 cells with 9-tandem repeat transfectants had significantly higher IL-6 levels than those with 7-tandem repeat transfectants (Figure 4B; 19.2 ± 3.6 vs. 6.4 ± 1.1 pg/mL, *p* = 0.014) and those with parental K562 cells (19.2 ± 3.6 vs. 7.1 ± 0.9 pg/mL, *p* = 0.017). When we compared the levels of IL-8 among parental K562 and L-SIGN 7-tandem repeat or 9-tandem repeat transfectants, we also found that K562 cells with 9-tandem repeat transfectants had significantly higher IL-8 levels than those with 7-tandem repeat transfectants (Figure 4C; 82.1 ± 24.5 vs. 35.9 ± 14.7 pg/mL) and those with parental K562 cells (82.1 ± 24.5 vs. 22.4 ± 0.7 pg/mL, *p* = 0.029).

## 3. Discussion

This study demonstrated that the DC-SIGN neck region showed almost all the 7/7 genotype (97.2%) in the study population, but the L-SIGN neck region had a wide variety of length polymorphisms, with 5–9 tandem repeats. The 9-tandem repeat allele of the L-SIGN neck region was significantly associated with the risk of DHF in the DEN-2 outbreak in Taiwan. Dengue patients carrying the 9-tandem repeat allele had a higher viral load and lower IFN-γ levels in the blood. This is the first report demonstrating that humans bearing the L-SIGN 9-tandem repeat allele are at higher risk of the development of DHF and tend to have depressed IFN-γ levels with higher viral loads once they are infected with DEN.

The East Asian population exhibits a notably elevated prevalence of the 9-tandem repeat allele within the L-SIGN neck variants, ranging between 11.8% and 13.8% (Table 2). By comparison, African and Caucasian population groups have a lower frequency, between 0% and 2.64% (Table 2). This finding holds significant interest, particularly given the high incidence of dengue infection in East Asia. Further validation across diverse populations experiencing dengue outbreaks in the future is warranted. The absence of the 8-tandem repeat allele in the studied population remains unexplained.

A previous study has investigated whether L-SIGN neck-region tandem repeats in heterozygous alleles or different tandem repeats are associated with the susceptibility and severity of certain infections [11]. The individuals with heterozygous L-SIGN neck-region repeats are more susceptible to SARS-CoV infection. In a large cohort study, it was demonstrated that the homozygous 7/7 genotype was significantly associated with an increased risk of HIV-1 infection and that the heterozygous 7/5 genotype correlated to resistance to HIV-1 infection [27]. A recent study performed in South Africa found no significant association for L-SIGN neck-region tandem repeats with susceptibility to tuberculosis infection [13] but no significant differences in the distributions of L-SIGN alleles and genotypes between HCV-infected patients and healthy controls; however, they observed that HCV-infected patients with 5-, 6-, and 7-repeat alleles had a higher HCV load when compared with 4- and 9-repeat allele carriers, suggesting that the VNTR may play a role in influencing HCV replication efficacy [26]. By contrast, we showed that the 9-tandem repeat allele carries a higher risk for DHF, and the heterozygous 7/6 genotype tends to be protective from DHF. Interestingly, the heterogeneity of the L-SIGN VNTR had no effect on DEN infection; the L-SIGN containing 5- or 7-repeats and transfected cells, 293T cells, might lead to a different outcome in our study [28].

The hallmark feature that distinguishes DHF from DF is the increase in vascular permeability or evidence of plasma leakage (i.e., pleural effusion, ascites, and/or hypoalbuminemia) that may, in turn, lead insidiously or rapidly to DSS. With regard to the expression spectra of different C-type lectins on different cells, DC-SIGN is expressed mainly on endocytic cells, such as dendritic cells and macrophages, whereas L-SIGN is expressed on endothelial cells in the liver and lymph nodes [29,30,31]. It has been reported that L-SIGN promoted West Nile virus infections much more efficiently than did DC-SIGN in mammalian cells [28]. The N-linked glycosylation site on either the ‘prM’ or E glycoprotein of flavivirus can bind to L-SIGN and mediate infection [32]. Indeed, we found that DHF patients had significantly higher AST and ALT levels and higher activated partial thromboplastin times (aPTTs), suggesting liver involvement of DEN infection in DHF. Genetic variations in L-SIGN in liver sinusoids or parenchyma may affect the DEN replication and immune responses, resulting in the coagulopathy and vascular leakage that are characteristic of DHF.

Studies of T-helper cytokine profiles in DEN patients have demonstrated that a shift from a Th1-dominant immune response to a Th2-biased response determined the disease progression to DHF and DSS [9,33]. It is known that DC-SIGN expression is positively regulated by the Th2 mediator, IL-4, but negatively regulated by the Th1 mediator, IFN-γ [34]. Although there is no evidence that L-SIGN expression is regulated by IL-4 or IFN-γ, we found that the L-SIGN neck-region 9-tandem repeat allele was associated with lower IFN-γ levels. This suggests that L-SIGN neck-region repeats may affect the lower IFN-γ production that upregulates DC-SIGN and/or L-SIGN expression(s) for DEN infection, perhaps to protect the virus from degradation or loss in the extracellular milieu in a vicious cycle. Thus, it is likely that individuals with the L-SIGN 9-tandem repeat allele (most heterozygotes) have less efficient viral degradation and, thereby, a higher viral load and altered immune response in dengue infections. DC-SIGN and its related protein, DC-SIGNR (L-SIGN), effectively block HIV budding from infected cells [35]. Whether the neck region of L-SIGN influences the viral life cycle and persistence of viruses within the host deserves further study. In addition, higher IL-6, IL-8, and MCP-1 levels had been found in K562 cells with 9-tandem repeat transfectants than in those with 7-tandem repeat transfectants and parental K562 cells, corresponding with previous clinical data from patients with severe DEN infections (Figure 3). Further studies are needed to differentiate whether the increased IL-6, IL-8, and MCP-1 levels resulted from increased replication or differences in signaling strength between L-SIGN with 7- and 9-tandem repeats. The spectrum of cytokines/chemokines induced by DEN-2-infected K562 cells is not limited to IL-6, IL-8, and MCP-1; additional studies are needed to investigate whether Th1/Th2 imbalance plays a role in L-SIGN associated with DHF pathogenesis (Figure 3).

This result, together with previous reports that L-SIGN neck-region repeat variants are associated with both HIV susceptibility and SARS-CoV pathogenesis [11,27], suggests that variations in L-SIGN may be of crucial importance in the outcomes of a number of infections due to L-SIGN-interacting pathogens. The fact that Asians, including Chinese, carry a higher frequency of neck-region 9-tandem repeats than do Caucasians suggests that protecting susceptible hosts from mosquito bites in dengue endemics or epidemic regions is essential until effective vaccines are available in Asia. Assessing the functional consequences of L-SIGN variants on the host’s immune responses against pathogens, including dengue, is necessary for developing a knowledge-based L-SIGN-pathway-targeted treatment. Hopefully, further worldwide studies concerning outbreaks involving other DEN serotypes will prove the association among these L-SIGN variants, immune responses, and severity of dengue infections.

## 4. Conclusions

In conclusion, this study demonstrated that DHF, a higher viral load, and lower IFN-γ levels were more frequently found in patients with the L-SIGN neck-region 9-tandem repeat allele.

## 5. Materials and Methods

### 5.1. Subjects

In a hospital-based case-control study, we have previously reported that patients with DHF were significantly associated with secondary DEN-2 infections [36,37], based on Th2 cytokine responses in patients from the 2002–2003 DEN-2 outbreak in Taiwan [38]. Employing decoded DNA samples from the same cohort of subjects, including 104 DHF, 109 DF, and 104 other febrile illness (OFI) patients, we investigated whether different forms of the CD209 and CD299 neck-region polymorphism were associated with DHF and immune responses. Study participants’ blood samples were drawn between one and three days after individual admission. This study was reviewed and approved by the Institutional Review Board of Chang Gung Memorial Hospital in Kaohsiung, Taiwan (Document No.: 92-005). DEN-2 infection was confirmed by clinical dengue symptoms and signs along with the detection of DEN-2 RNA by quantitative RT-PCR or the detection of IgM to DEN in the blood [36,38]. The diagnosis of DHF was made according to the criteria of the World Health Organization for DHF, which include thrombocytopenia (<100,000/mm^3^), hemorrhagia, and evidence of plasma leakage, reflected by hemoconcentration (≥20%), pleural effusion, ascites, and/or hypoalbuminemia [20,21]. Patients with OFI were defined as having febrile illness with no detectable DEN-specific IgM, no detectable DEN RNA, and no obvious or reported bacterial etiology for their illness during the same study period. Plasma levels of IFN-α, IFN-γ, IL-10, and IL-13 were measured using ELISA kits manufactured by Bender MedSystems, Inc., Vienna, Austria. The results were calculated from the interpolation of a standard curve made from a series of known concentrations of commercial standards.

### 5.2. Variable-Number Tandem Repeat and Genotyping

Genomic DNA was extracted from whole blood. Because of the high sequence-identities of *CD209* and *CD299*, special care was taken to design primers that specifically amplified the neck regions of both genes. The *CD209* and *CD299* variable-number tandem repeats in exon 4 were polymerase chain reaction (PCR)-amplified from genomic DNA using the following primers: forward 5′-CCA CTT TAG GGC AGG AC-3′ and reverse 5′-AGC AAA CTC ACA CCA CAC AA-3′ for DC-SIGN and 5′-AGG GCT TGG CAC ACA GTA GGT G-3′ and reverse 5′-ACC CTT GAT GTG CAG GAA CT-3′ for L-SIGN. PCR amplifications were performed in a final volume of 25 μL containing 30 ng of genomic DNA, 10 nM primers, 10 mM dNTP, 25 mM MgCl_2_, and 0.5 U of Taq polymerase (YEA, Randolph, MA, USA). The cycling conditions were as follows: 5 min at 94 °C, followed by 30 cycles for 15 s at 95 °C, 7 s at 61 °C, and 30 s at 72 °C, and final extension at 72 °C for 7 min for *CD209*; 5 min at 94 °C, followed by 30 cycles for 30 s at 94 °C, 30 s at 64.2 °C, and 30 s at 72 °C, and final extension at 72 °C for 5 min for *CD299*. Neck-region tandem repeat alleles were distinguished by fragment lengths of polymorphisms after agarose gel electrophoresis and ethidium bromide staining (The sizes of the PCR products for *CD299* with 7-tandem repeat or 9-tandem repeat alleles are 680 b.p. and 820 b.p.). In a pilot study, the lengths of different representative alleles (bands) of PCR products were confirmed by direct sequencing to ensure specific amplifications of *CD209* and *CD299* neck-region tandem repeat alleles. We only included studies that involved the presentation of the *CD299* neck-region polymorphism in different populations/countries and summarized these results in Table 2.

### 5.3. Preparation of DEN-2 Virus

DEN-2 (New Guinea C strain) viruses were propagated in *Aedes albopictus* C6/36 cells, as previously described [38]. Virus titers were determined by a standard plaque-forming assay on BHK-21 cells and adjusted to 2 × 10^7^ PFU/mL in RPMI 1640 (Gibco BRL, Grand Island, NY, USA) with 10% fetal calf serum (FCS, Gibco BRL) in a large-scale preparation. The same batch of viruses was collected and stored at −80 °C before use. BHK-21 cells (2 × 10^5^/mL) suspended in modified Eagle’s medium were seeded into each 24-well culture plate (Nalge Nunc, Rochester, NY, USA) and incubated at 37 °C in a humidified CO_2_ incubator. The cells were cultured overnight before viral adsorption. A series of viral dilutions made in the culture medium at 0.1 mL was adsorbed onto a monolayer of BHK-21 cells for 1 h at 28 °C. Each virus-infected culture well was overlaid with 0.8 mL of 1.5% carboxymethyl cellulose containing 2% FCS for an additional 6 days. The plaque-forming wells were fixed with 10% formalin for 60 min, followed by staining with 0.5% crystal violet in normal saline solution containing 50% alcohol and 5% formalin. The results were calculated by counting plaques in the four replicate wells, and the final results were corrected by individual dilution factors.

### 5.4. Preparation of L-SIGN Constructs with Neck 7- or 9-Tandem Repeats

The wild-type L-SIGN cDNA with neck 7-tandem repeats (N7) in pcDNA3.1 (Invitrogen, Inc., Carlsbad, CA, USA) was obtained from the original plasmid, pUNO-hDCSIGN2a (InvivoGen, Inc., San Diego, CA, USA). To make an L-SIGN construct with a 9-tandem-repeat allele, the entire exon 4 harboring nine-tandem repeats (N9) was amplified by 3-RACE and nest PCR from complementary DNA from a human carrying the N9 allele. From the 5’ end in exon 1 to the 3’ end of the L-SIGN neck region, the pGlow-TOPO-N9 plasmid was cut and replaced into the pcDNA3.1 plasmid (N9 constructs) using *KpnI* and *XbaI* restriction enzymes (New England Biolabs, Inc., Ipswich, MA, USA) and *T4* ligase (New England Biolabs, Inc., Ipswich, MA, USA). The plasmid DNA was extracted and subjected to DNA sequencing to ensure that the N7 and N9 constructs had a 2-tandem repeat difference.

### 5.5. Transfection of N7 and N9 L-SIGN Constructs into Cell Lines

U937 and K562 cells were maintained in RPMI 1640 supplemented with 10% FBS, 10 units/mL of penicillin, and 10 μg/mL of streptomycin, unless otherwise stated. The plasmid with the N7 L-SIGN constructor was transfected into U937 cells and K562 cells using nucleofector (Lonza) according to the manufacturer’s instructions. Stable clones of U937 and K562 cells revealing N7 L-SIGN expression were selected with 400 μg/mL of G418 for 2 to 3 weeks. Cells with L-SIGN mRNA expression and cell-surface expression of L-SIGN, verified respectively by flow cytometry and RT-PCR, were subjected to functional validation of the receptor responsible for DEN infection. L-SIGN was detected using a flow cytometer and anti-L-SIGN (FAB162P, R&D) antibodies. The cells were washed with PBS twice and followed by incubation with antibodies at a 1/50 dilution for 30 min at 4 °C. The cells were washed and fixed with paraformaldehyde prior to FACS analysis (BD Biosciences) and data processing with CellQuest software version 4.02 (Becton Dickinson). For RT-PCR detection, cellular RNAs were harvested with Trizol™ (Invitrogen, Waltham, MA, United States) and transferred to cDNA by oligo dT and MMLV reverse transcriptase. The PCR was amplified using the forward primer 5′-CAACAACCAGTGGCATCAGA-3′ and the reverse primer 5′-GGCCATGTATCTGCTGGAAT-3′. The PCR reaction mixture (final volume: 20 μL) contained 2 μL of cDNA, 1 μL of 10 μM forward primer, 1 μL of 10 μM reverse primer, 10 μL 2× of SYBR Green PCR Master Mix, and 6 μL of H_2_O. The PCR reactions were performed in triplicate. The relative quantification values for the target gene expression were calculated from the accurate Ct value, which is the PCR cycle at which an increase in reporter fluorescence from the SYBR Green dye can be first detected above the baseline signal. To calculate the fold induction of the *CD299* mRNA in the cells, the averaged ΔCt values calculated for the control cells were subtracted from the ΔCt values calculated for the *CD299*-transfected cells to calculate ΔΔCt. Then, the fold induction for each well was calculated using the 2^−(ΔΔCt)^ formula. After demonstrating ectopic L-SIGN expression on myeloid cells, we further transfected the N7 and N9 constructs into K562 cells for comparing whether N9 mediated different virus replication and immune responses from N7-mediated ones.

### 5.6. Proinflammatory Cytokines, Chemokines, and Viral Replication in K562 Cells

To measure cytokine/chemokine production, K562 transfectant cells with N7 and N9 L-SIGNs were seeded into 24-well tissue-culture plates in triplicate and incubated at 37 °C in a humidified 5% CO_2_ atmosphere in the presence or absence of DEN-2 (MOI = 5). K562 cells were incubated with the virus at 37 °C for 1 h, and the unbound virus was removed by two washes with phosphate-buffered saline (PBS; pH 7.4). After 4 h, culture supernatants were collected and assayed for MCP-1 (eBioscience, Inc., San Diego, CA, USA), IL-6 (R&D Systems, Minneapolis, MN, USA), and IL-8 (R&D Systems, Minneapolis, MN, USA) concentrations, using ELISA according to the manufacturer’s recommendations. The results were calculated by interpolation from a standard curve made from a series of well-known concentrations of standards [37,39]. The RNA samples extracted from the supernatants and cellular parts of the parental K562 cells or N7 and N9 L-SIGN transfectants at 4 h or 48 h post infection with DEN-2 were stored at –80 °C until further use, as previously described [36]. The viral load was measured by real-time RT-PCR using an ABI 7500 quantitative PCR machine (Applied Biosystems, PerkinElmer, Foster City, CA, USA) for 40 cycles and TaqMan technology [36]. The forward primer, reverse primer, and nested fluorescent probe sequence for detecting DEN-2 were 5′-GGCTTAGCGCTCACATCCA-3′, 5′-GCTGGCCACCCTCTCTTCTT-3′, and FAM-CGCCCACCACTATAGCTGCCGGA-TAMRA, respectively.

### 5.7. Statistical Analyses

Genotypes and allele frequencies in different groups were compared by gene counting and chi-squared analysis or Fisher’s exact probability test, depending on the allele frequency. The odds ratio and 95% CI were used to measure the strength of the associations in the genetic risk association study. For all in the vitro studies, statistical significance was calculated using Student’s two-tailed *t*-test. A *p*-value of less than 0.05 indicated statistical significance. Data were calculated and analyzed using SPSS (version 13.0).

## Figures and Tables

**Table 1:** Demographic data of patients with DHF or DF.

	DHF N = 104	DF N = 109	*p* Value
Characteristics			
Gender, Male/Female	47/57	48/61	0.865
White Blood Cell Count (cells/mm^3^)	4374 ± 238	4130 ± 190	0.425
Hemoconcentration *	38/104 (37%)	0/109 (0%)	<0.001
Platelet Count (×10^4^/mm^3^)	2.58 ± 0.28	10.93 ± 0.71	<0.001
Serum Albumin Level (g/dL)	3.06 ± 0.08	3.67 ± 0.21	0.001
Serum AST Level (U/mL)	313.8 ± 74.6	70.1 ± 8.1	0.002
Serum ALT Level (U/mL)	142.7 ± 21.9	67.1 ± 11.3	0.003
Secondary infection rate	80/104 (77%)	8/109 (7%)	<0.001
Pleural Effusion ^#^	57/75 (76%)	0/59 (0%)	<0.001
Ascites ^#^	47/85 (55%)	1/68 (2%)	<0.001
Values are the mean ± SE or number.			

* The mean hematocrit for adult healthy controls is 37%. A cutoff value of ≥44% of hematocrit was chosen as evidence of hemoconcentration, which is approximately 20% above the mean for the population. Primary and secondary dengue infections are defined by detectable DEN-specific IgM and IgG, respectively, within one week of the illness. ^#^ Positive/total case numbers.

**Table 2:** Distributions of L-SIGN neck-region alleles in different populations.

Allele Frequency	Taiwan (This Study) N = 317	East Asian (Barreiro and Quintana-Murci, 2006 [25]) N = 251	Chinese, Hong Kong (Chan et al., 2006 [11]) N = 380	African (Barreiro et al., 2007 [13]) N = 360	Caucasian (Nattermann et al., 2006 [26]) N = 100
9	12.9%	11.8%	12.0%	2.6%	2.5%
8	0%	0.2%	0%	1.0%	0%
7	68.5%	69.7%	69.7%	59.7%	50.5%
6	3.8%	2.6%	4.9%	24.2%	12.0%
5	14.8%	15.5%	0%	11.8%	30.5%
4	0%	0%	0%	0.7%	4.5%

**Table 3:** Allelic frequency distributions of L-SIGN neck region in patients in DHF, DF, and OFI groups.

	DHF	DF	OFI	DF	DHF		OFI	DHF	
Allele	N = 208	N = 218	N = 208	Reference	OR (95% CI)	*p* Value	Reference	OR (95% CI)	*p* Value
9	41 (20%)	21 (10%)	20 (10%)	1	2.30 (1.31–4.05)	0.004	1	2.31 (1.30–4.10)	0.004
7	132 (64%)	155 (71%)	147 (71%)	1	0.71 (0.48–1.06)	0.093	1	0.72 (0.48–1.09)	0.118
6	4 (2%)	8 (4%)	12 (6%)	1	0.52 (0.15–1.74)	0.284	1	0.32 (0.10–1.01)	0.052
5	31 (15%)	34 (16%)	29 (14%)	1	0.95 (0.56–1.61)	0.843	1	1.08 (0.63–1.87)	0.780

**Table 4:** Genotypic frequency distributions of L-SIGN neck region in patients in DHF, DF, and OFI groups.

	DHF	DF	OFI	DF	DHF		OFI	DHF	
Genotype	N (%)	N (%)	N (%)	Reference	OR (95% CI)	*p* Value	Reference	OR (95% CI)	*p* Value
9/9	5 (5%)	3 (3%)	0 (0%)	1	1.79 (0.42–7.66)	0.436	1	NA	0.996
9/7	22 (21%)	13 (12%)	16 (15%)	1	1.98 (0.94–4.18	0.073	1	1.48 (0.73–3.00)	0.283
9/6	2 (2%)	0 (0%)	2 (2%)	1	NA	0.996	1	1.00 (0.14–7.24)	1.000
9/5	7 (7%)	2 (2%)	2 (2%)	1	3.86 (0.78–19.03)	0.097	1	3.68 (0.75–18.15)	0.110
7/7	46 (44%)	58 (53%)	51 (49%)	1	0.70 (0.41–1.20	0.191	1	0.82 (0.48–1.42)	0.487
7/6	1 (1%)	7 (6%)	9 (9%)	1	0.14 (0.02–1.17)	0.070	1	0.10 (0.01–0.82)	0.032
7/5	17 (16%)	19 (17%)	20 (19%)	1	0.93 (0.42–1.90)	0.833	1	0.82 (0.40–1.67)	0.587
6/5	1 (1%)	1 (1%)	1 (1%)	1	10.49 (0.07–16.99)	0.973	1	1.00 (0.06–16.20)	1.000
5/5	3 (3%)	6 (6%)	3 (3%)	1	0.51 (0.12–2.09)	0.350	1	1.00 (0.20–5.07)	1.000

## Data Availability

The data presented in this study are available on request from the corresponding author.
